# Predictors of Adolescents’ Response to a Web-Based Intervention to Improve Psychosocial Adjustment to Having an Appearance-Affecting Condition (Young Person’s Face IT): Prospective Study

**DOI:** 10.2196/35669

**Published:** 2023-01-18

**Authors:** Deniz Zelihić, Kristin J Billaud Feragen, Are Hugo Pripp, Tine Nordgreen, Heidi Williamson, Johanna Kling

**Affiliations:** 1 Centre for Rare Disorders Rikshospitalet Oslo University Hospital Oslo Norway; 2 Department of Psychology Faculty of Social Sciences University of Oslo Oslo Norway; 3 Oslo Centre of Biostatistics and Epidemiology Research Support Services Oslo University Hospital Oslo Norway; 4 Division of Psychiatry Haukeland University Hospital Bergen Norway; 5 Centre for Appearance Research University of the West of England Bristol United Kingdom

**Keywords:** visible difference, web-based interventions, eHealth, body esteem, social anxiety, adolescents

## Abstract

**Background:**

Adolescents with a condition affecting their appearance that results in a visible difference can be at risk of psychosocial distress and impaired adjustment. Evidence for the effectiveness of existing interventions in improving psychosocial outcomes is limited, and relevant treatment can be difficult to access. Young Person’s Face IT (YPF), a self-guided web-based intervention, has demonstrated potential in reducing social anxiety in adolescents with a visible difference. However, more knowledge is needed about the variables that contribute to variations in intervention effects to identify those who may benefit most from YPF.

**Objective:**

This study aimed to investigate demographic, psychosocial, and intervention-related variables as predictors of overall intervention effects after adolescents’ use of YPF.

**Methods:**

We used longitudinal data collected as part of a larger, ongoing mixed methods project and randomized controlled trial (ClinicalTrials.gov NCT03165331) investigating the effectiveness of the Norwegian version of YPF. Participants were 71 adolescents (mean age 13.98, SD 1.74 years; range 11-18 years; 43/71, 61% girls) with a wide range of visible differences. The adolescents completed primary (body esteem and social anxiety symptoms) and secondary (perceived stigmatization, life disengagement, and self-rated health satisfaction) outcome measures at baseline and postintervention measurement. The predictor variables were demographic (age and gender), psychosocial (frequency of teasing experiences related to aspects of the body and appearance as well as depressive and anxiety symptoms), and intervention-related (time spent on YPF) variables.

**Results:**

Two-thirds (47/71, 66%) of the adolescents completed all YPF sessions and spent an average of 265 (SD 125) minutes on the intervention. Backward multiple regression analyses with a 2-tailed *P*-value threshold of .20 revealed that several variables were retained in the final models and predicted postintervention outcome changes. Body esteem was predicted by age (*P*=.14) and frequency of teasing experiences (*P*=.09). Social anxiety symptoms were predicted by gender (*P*=.12), frequency of teasing experiences (*P*=.03), depressive and anxiety symptoms (*P*=.08), and time spent on YPF (*P*=.06). Perceived stigmatization was predicted by age (*P*=.09), gender (*P*=.09), frequency of teasing experiences (*P*=.19), and depressive and anxiety symptoms (*P*=.06). Life disengagement was predicted by gender (*P*=.03), depressive and anxiety symptoms (*P*=.001), and time spent on YPF (*P*=.14). Self-rated health satisfaction was predicted by age (*P*=.008). However, the results were limited by relatively low explained postintervention variance, ranging from 1.6% to 24.1%.

**Conclusions:**

This study suggests that adolescent boys, adolescents who experience higher levels of psychosocial distress related to their visible difference, and adolescents who spend sufficient time on YPF may obtain better overall intervention effects.

## Introduction

### Background

Physical appearance can be a source of psychological and social distress, especially during adolescence. A heightened focus on appearance and pressures to conform to appearance ideals may negatively affect adolescents’ psychological health in terms of anxiety, depression, and low self-esteem [1]. Moreover, negative peer influences, including teasing related to appearance and weight, may put adolescents at particular risk of psychosocial distress, including body dissatisfaction [2]. Consequently, having an appearance that is not accordant with societal norms may make some adolescents particularly vulnerable to appearance concerns and stigmatizing experiences [3,4].

A range of congenital and acquired conditions may affect facial and bodily appearances and lead to what is referred to as a visible difference [5]. Congenital conditions may include craniofacial anomalies (eg, cleft lip and palate or differences of sex development) and skin conditions (eg, eczema or psoriasis) [5,6]. Acquired conditions may result from medical interventions (eg, hair loss from radiation therapy) or accidental traumas (eg, traffic injuries and burn scars). Prevalence rates of those living with a visible condition are uncertain, although estimations show that 2.27% of individuals have a notable congenital or acquired visible difference, with a significant yearly incidence of an acquired visible difference [7].

### Demographic and Psychosocial Influences on Adolescents’ Adjustment to a Visible Difference

Some of the main challenges encountered by many adolescents with a visible difference include experiences of being stared at and questioned about their appearance by others [3] and teased or bullied by peers because they look different [8-10]. Although characteristics associated with a person’s visible difference (eg, size or shape of facial or bodily features) poorly predict psychological well-being and general psychological adjustment [11,12], some studies suggest that age may play a role in experiences of having a visible difference. As children transition into adolescence, they may become increasingly aware of their condition and how it affects their appearance [13]. Longitudinal findings also show that experiences of teasing during adolescence may have a negative impact on appearance satisfaction and emotional well-being [14]. In addition, more frequent stigmatization has been predicted by higher age in children, as reported by their parents [3].

Experiences of teasing can negatively influence adolescents’ self-esteem and lead some to choose behavioral avoidance—refraining from engagement in social activities—as a coping strategy for fear of being teased for their difference [9,10] or negatively evaluated by others [15]. Moreover, studies show that stigmatizing experiences attributable to a visible difference can negatively influence adolescents’ psychological adjustment and health-related quality of life [16]. It is therefore not surprising that adolescents with a visible difference, irrespective of type and severity, may report increased symptoms of anxiety compared with unaffected peers [17]. These findings underscore that challenging peer interactions, and particularly those of a stigmatizing nature, may be especially impactful on psychosocial well-being during adolescence.

It is generally recognized from adolescent community samples that girls tend to experience lower body esteem than boys [18] and also report greater disengagement in activities such as school attendance and spending time with friends and family [19]. There is also evidence suggesting gender differences within samples affected by a visible difference [4,8,20]; for instance, studies have found higher levels of emotional and social challenges [21], higher levels of anxiety [20], and lower appearance satisfaction in girls than in boys [4,8].

### Available Support to Promote Adolescents’ Adjustment

Psychosocial support for adolescents with a visible difference has typically been based on an eclectic approach and includes a wide range of therapeutic approaches and techniques such as social skills training (SST) as well as techniques based on cognitive behavioral therapy (CBT), psychoeducation, mindfulness, and acceptance and commitment therapy [22]. Psychosocial interventions incorporating techniques based on SST and CBT have specifically shown potential in improving psychosocial well-being and promoting adjustment in adolescents challenged by their visible difference [23,24] and may be offered as an alternative or adjunct to biomedical support [5].

Internet-delivered CBT (ICBT), which has shown intervention effects on mental health outcomes that are comparable with those shown by standard face-to-face CBT [25], could also offer several benefits for adolescents experiencing challenges related to their visible difference; for instance, because raising appearance issues face to face with health care professionals may be experienced as too personal and difficult [26], some adolescents could favor more easily accessible support that offers a greater degree of anonymity and confidentiality when discussing appearance concerns [15].

### Young Person’s Face IT

To date, Young Person’s Face IT (YPF) is the only self-guided internet-based intervention using a web-based platform developed for adolescents with a visible difference. YPF was developed at the Centre for Appearance Research based at the University of the West of England, Bristol, United Kingdom, in close collaboration with adolescents with a visible difference and their parents, clinical experts, and health professionals [24,27]. The therapeutic content is based on SST and CBT techniques and consists of 7 weekly sessions and 1 booster session completed 6 weeks later to maintain the therapeutic effect [27]. Each session is completed independently and is intended to take approximately 30 to 40 minutes. The sessions provide advice and guidance in written, audio, and video formats on how to adjust to common challenges related to having a visible difference and encourage adolescents to practice strategies to manage staring, bullying, and anxiety through interactive and homework activities (for a more detailed description of the intervention, refer to the study by Williamson et al [27]).

The feasibility and acceptability of YPF has been explored in several countries [24,28-30] (Feragen, K, unpublished data, September 2016), and a smaller feasibility trial found increased postintervention levels of body esteem and lower levels of social anxiety in adolescents who completed YPF, compared with a control group [24]. Intervention engagement (defined as the number of YPF sessions completed) was found to be a contributory factor in the smaller feasibility trial, with higher number of sessions completed predicting a positive intervention effect [24]. The effectiveness of YPF in improving body esteem and reducing symptoms of social anxiety, perceived stigmatization, and life disengagement was also recently evaluated in a randomized controlled trial (RCT) [31]. The RCT showed that adolescents in the intervention group had significantly lower levels of social anxiety than the control group at the postintervention measurement. The RCT also indicated a gender difference, showing that the intervention response to YPF seemed to be stronger for boys than for girls regarding social anxiety and life disengagement [31].

### Study Objectives

The objective of this exploratory study was to contribute to the accumulating body of research on the effectiveness of YPF in promoting adolescents’ adjustment to their visible difference [24,28-31] by further investigating which adolescents are likely to benefit from the intervention. On the basis of knowledge about variables of importance from previous research, we specifically investigated whether demographic (age and gender), psychosocial (frequency of teasing experiences as well as depressive and anxiety symptoms), and intervention-related (time spent on YPF) variables predict changes in body esteem and social anxiety (primary outcomes) in adolescents with a visible difference after completion of YPF. We also investigated whether these variables predict changes in perceived stigmatization, life disengagement, and self-rated health satisfaction (secondary outcomes).

## Methods

### Study Design

This study was conducted at the Centre for Rare Disorders based at Oslo University Hospital and used longitudinal data collected as part of a larger, ongoing mixed methods project and RCT (ClinicalTrials.gov NCT03165331) investigating the effectiveness of the Norwegian version of YPF.

### Ethics Approval

The study was reviewed by the Regional Committee for Medical Research Ethics South East Norway (reference number: 2015/2440) and accepted by the hospital’s Data Protection Office. All participants provided signed consent before enrollment. For participants aged <16 years, consent was also obtained from both primary caregivers.

### Procedure

Participants were recruited nationwide between April 2019 and February 2021 from university hospitals, specialist treatment units, local health care services, and patient organizations, as well as through social media platforms [32]. Participants and participants’ primary caregivers contacted the research team by telephone or email if they wished to participate in the study. After initial contact, all participants (and parents if the adolescent was aged <16 years) were contacted via telephone by the research team and were screened for eligibility. The inclusion criteria were as follows: (1) age between approximately 12 and 17 years with a visible difference and self-identified appearance-related distress, as well as experiences of teasing and bullying; (2) access to the internet and a home computer or tablet device; (3) minimum reading level corresponding to that of someone aged 12 years; and (4) normal or corrected-to-normal vision. The exclusion criteria were as follows: (1) a diagnosis of clinical depression, psychosis, eating disorder, or posttraumatic stress disorder or within 12 months of traumatic injury (as participants with these conditions were considered to require other higher-level or intensive interventions); (2) learning disabilities that would impede understanding of the intervention content; and (3) currently receiving a psychological face-to-face intervention.

Before being randomized to an intervention group receiving YPF or to a waiting list control group in the larger mixed methods project and RCT, participants completed outcome measures (baseline). Participants in the control group received the intervention (YPF) 13 weeks after the intervention group and completed outcome measures before starting the intervention. Participants from both groups completed outcome measures 13 weeks after completing YPF (postintervention measurement). In this study, all participants who had completed YPF (from both the intervention group and the waiting list control group) were included. Outcome measures were administered through a secure web-based data collection platform (Service for Sensitive Data).

### Participants

We assessed 137 participants for eligibility, of whom 102 (74.5%) were randomized (n=6, 4.4%, were excluded for not meeting inclusion criteria; and n=29, 21.2%, were excluded because they changed their minds or did not respond after initial screening for eligibility). Of these 102 participants, we excluded 31 (30.4%) because of missing postintervention data. Of the 71 participants remaining, 1 (1%) was identified as an outlier, and 1 (1%) did not start the intervention; both were removed from the data set. Participants who were excluded because of missing postintervention data and participants included in the study did not significantly differ in age (*t*_100_=0.697; *P*=.49), gender (*χ*^2^_1_=1.9; *P*=.16), frequency of teasing experiences related to body shape or weight (*t*_100_=–0.222; *P*=.83), or depressive and anxiety symptoms (*t*_99_=–0.587; *P*=.56).

The final sample included 71 participants (n=43, 61% girls) aged 11 to 18 years (mean 13.98, SD 1.74, years) with a wide range of conditions leading to a visible difference. Demographic characteristics are presented in detail in Table 1.

**Table 1:** Demographic characteristics of participants by gender and for the total sample.

Variable		Boys	Girls	Total sample
Age (years), mean (SD)		13.89 (1.73)	14.05 (1.77)	13.99 (1.74)
**Type of visible difference (boys: n=28, girls: n=43, total sample: N=71), n (%)**				
	Craniofacial condition	19 (68)	30 (70)	49 (69)
	Condition affecting body form	4 (14)	7 (16)	11 (16)
	Skin condition	4 (14)	4 (9)	8 (11)
	Acquired condition	1 (4)	1 (2)	2 (3)
	Unspecified condition	N/A^a^	1 (2)	1 (1)
**Fathers’ educational level^b^ (boys: n=27, girls: n=40, total sample: N=67), n (%)**				
	High	12 (44)	12 (30)	24 (36)
	Medium	8 (30)	15 (38)	23 (34)
	Low	6 (22)	7 (17)	13 (20)
	Unspecified	1 (4)	6 (15)	7 (10)
**Mothers’ educational level (boys: n=28, girls: n=40, total sample: N=68), n (%)**				
	High	12 (43)	16 (40)	28 (41)
	Medium	15 (53)	17 (42)	32 (47)
	Low	1 (4)	1 (3)	2 (3)
	Unspecified	N/A	6 (15)	6 (9)

^a^N/A: not applicable.

^b^Parents’ educational level was obtained based on information related to their occupation; for example, an academic position can be expected to correspond to a high educational level, a position within the general support system (eg, school personnel or health care professional) can be expected to correspond to a medium educational level, and a position as a skilled laborer can be expected to correspond to a low educational level.

### Measures

#### Outcomes

##### Body Esteem

The Body Esteem Scale for Adolescents and Adults (BESAA), specifically the appearance esteem subscale (appearance esteem subscale of the BESAA [BE-Appearance]), assessed body esteem [33]. The subscale contains 10 items rated on a 5-point Likert scale ranging from 0 (*never*) to 4 (*always*). Statements include “I worry about the way I look” and “I look as nice as I’d like to.” After negatively worded items have been reversed, higher mean values indicate higher levels of appearance esteem. The BESAA has shown good psychometric properties among adolescent community samples [18] and among adolescents with a visible difference [34]. In this study, for the BE-Appearance subscale, Cronbach α values were .89 for boys and .95 for girls.

##### Social Anxiety

The Social Anxiety Scale for Adolescents (SAS-A) assessed experiences of social anxiety [35]. The SAS-A contains 22 items (4 filler items not included in calculations) divided into 3 subscales that are rated on a 5-point scale ranging from 1 (*never*) to 5 (*always*). The first subscale, fear of negative evaluation (fear of negative evaluation [SAS-A subscale; FNE]), contains 8 items (eg, “I worry about being teased”). The second subscale, social avoidance and distress specific to new situations or unfamiliar peers (social avoidance and distress specific to new situations (SAS-A subscale [SAD-New]), includes 6 items (eg, “I feel shy around people I don’t know”). The third subscale, social avoidance and distress in general (social anxiety and distress in general [SAS-A subscale; SAD-General]), contains 4 items (eg, “It’s hard for me to ask others to do things with me”). A total scale score is also computed based on 18 items. Higher scores indicate higher levels of social anxiety. The SAS-A has shown good psychometric properties among adolescent community samples [36]. In this study, Cronbach α was calculated for all subscales as well as the total scale (FNE: α=.95 for boys and α=.92 for girls, SAD-New: α=.89 for boys and α=.89 for girls, SAD-General: α=.77 for boys and α=.84 for girls, and total scale: α=.95 for boys and α=.93 for girls).

##### Perceived Stigmatization

The Perceived Stigmatization Questionnaire (PSQ) measured perceptions of stigmatization behaviors [37]. The PSQ consists of 21 items divided into 3 subscales that are rated on a 5-point Likert scale from 1 (*never*) to 5 (*always*). The subscales evaluate the absence of friendly behavior (absence of friendly behavior [PSQ subscale; AFB]), experiences of confused and staring behaviors from others (confused and staring behaviors [PSQ subscale; CSB]), and the extent to which respondents encounter hostile behavior (hostile behavior [PSQ subscale; HB]). A total scale score is also computed based on all items. Examples of items include “Strangers are polite to me,” “People do not know what to say to me,” and “People laugh at me.” After positively worded items are reversed, higher scores indicate higher levels of perceived stigmatization. One item in the PSQ (“People are nice to me”) was omitted from the measure because of human error. This error was accounted for when calculating the AFB subscale and total scale scores. The PSQ has shown acceptable psychometric properties among adolescents with a visible difference [38] and has been previously translated and used with Dutch adults with a visible difference [39]. In this study, Cronbach α was calculated for all subscales as well as the total scale (AFB: α=.68 for boys and α=.75 for girls, CSB: α=.69 for boys and α=.68 for girls, HB: α=.89 for boys and α=.87 for girls, and total scale: α=.87 for boys and α=.87 for girls).

##### Life Disengagement

The Body Image Life Disengagement Questionnaire (BILD-Q) assessed the extent to which appearance-related worries affect engagement or intention to engage in different life activities (eg, “Going to a social event” and “Spend time with friends and family”) [19,40]. The current BILD-Q [19] consists of 9 items rated on a 4-point Likert scale from 1 (“Hasn’t stopped me at all”) to 4 (“Stopped me all the time”); a previous 10-item version of the BILD-Q was used in this study [40]. Higher scores indicate higher levels of life disengagement. The BILD-Q has shown acceptable psychometric properties in an adolescent community sample [19]. In this study, Cronbach α values were .76 for boys and .87 for girls for the total scale.

##### Health-Related Quality of Life

The EQ-5D-5L questionnaire [41] was used to measure self-rated health satisfaction in 5 dimensions: mobility (eg, “I have no problems in walking about”), self-care (eg, “I have no problems with washing or dressing myself”), usual activities (eg, “I have no problems doing my usual activities”), pain or discomfort (eg, “I have no pain or discomfort”), and anxiety or depression (eg, “I am not anxious or depressed”). Each dimension is rated on 1 of 5 different levels (“no problems,” “slight problems,” “moderate problems,” “severe problems,” and “unable to”). For the purpose of this study, participants’ ratings on the 5 health dimensions are descriptively reported in the *Results* section, with only the dimension of anxiety or depression included as a predictor in our analyses. Participants also self-rated their health satisfaction from 0 (“the worst health you can imagine”) to 100 (“the best health you can imagine”) on the EQ-5D-5L questionnaire's visual analog scale (EuroQol visual analog scale [EQ-VAS]), which was used as a secondary outcome in the analyses.

#### Predictors

##### Experiences of Appearance-Related Teasing

We assessed the frequency of teasing experiences and subsequent distress with 2 items drawn from Project EAT-III [42]. The items assessed frequency of teasing experiences related to “weight and shape” and “the way you look” and feelings of distress from teasing related to “weight and shape” and “the way you look.” Distress was scored on a 5-point scale (*not upset* to *very upset*), with higher scores reflecting greater distress. To reduce the number of variables that would be included in our analyses, correlations between frequency of teasing experiences about body form and weight and teasing experiences about appearance were calculated and showed a moderately strong positive association (*r*=0.475; *P*<.001). Correlations between distress experienced from teasing about body form and weight and teasing about appearance were also calculated and showed a strong positive association (*r*=0.670; *P*<.001). Hence, we computed 2 single variables assessing frequency of teasing experiences and teasing-related distress. Cronbach α was calculated for both computed variables (frequency of teasing experiences in general: α=.59 for boys and α=.63 for girls and general teasing-related distress: α=.70 for boys and α=.83 for girls).

##### Engagement With YPF

Engagement was measured in 2 different ways: by the number of sessions completed and by calculating mean time (in minutes) spent on YPF. The YPF program automatically records time spent on each session for each participant. Still, recorded time may not always represent actual time use because participants might forget to log out, which leads to nonvalid measurement of time spent on a particular session. However, each session consists of several subsessions, and time spent is also recorded for each subsession. Therefore, to control for possible errors of total time spent and to obtain a more precise measure of time, we inspected participants’ time use on each subactivity within each session. When unrealistic time use was suspected for any given subactivity within a given session, we calculated a mean based on those subactivities that had representative time use and replaced the suspected time with this mean.

#### Translations

The BESAA, SAS-A, PSQ, and BILD-Q had not been translated into Norwegian before the project. The BESAA, SAS-A, and PSQ were translated following recommended procedures, including back translation [43]. Back translation was not performed for the BILD-Q measure.

### Statistical Analysis

All analyses were conducted with SPSS software (version 26.0; IBM Corp) and included preliminary and main analyses. Difference scores were calculated based on baseline and postintervention scores for primary and secondary outcomes and used in all analyses to assess the degree of change associated with YPF. Preliminary analyses included inspection of missing data and outliers, as well as a descriptive exploration of participants’ intervention completion, frequency of teasing experiences and related distress, self-rated health states, baseline and postintervention means, and bivariate correlations between prognostic variables and primary and secondary outcomes. The strengths of associations were interpreted using the guidelines formulated by Cohen [44], defined as weak (.10), moderate (.30), and strong (.50) relationships. Results from the preliminary analyses are presented by gender (except for results on missing data and outliers) and for the total sample (except for the bivariate correlations).

Main analyses were conducted to investigate predictors of intervention effects. This included identification of predictors following recommended procedures [45,46] using backward multiple regression analyses with a 2-tailed *P-*value threshold of .20. All regression models were evaluated using adjusted *R*^2^ (*R*^2^_adjusted_). Demographic (age and gender), psychosocial (frequency of teasing experiences as well as depressive and anxiety symptoms), and intervention-related (time spent on YPF) variables were entered in all regression models. Body esteem and social anxiety were defined as primary outcomes. Secondary outcomes were perceived stigmatization, life disengagement, and self-rated health satisfaction (ie, EQ-VAS), selected to evaluate how YPF affects other influential aspects of adolescents’ adjustment to a visible difference. A 95% CI (2-tailed) was used in all main analyses. Listwise deletion was used to handle missing data for the main analyses, and pairwise deletion was used for the bivariate correlations. Owing to the exploratory nature of this study, no adjustment for multiple comparisons was made [47]. Results from the main analyses are only presented for the total sample because gender was included as a predictor in the regression models.

## Results

### Preliminary Analyses

#### Overview

Rates of missing data for the psychosocial and intervention-related variables were small (baseline depressive and anxiety symptoms: 1% and time spent on YPF: 3%). There were no missing data for the primary and secondary outcomes, except for 1% missing for the BILD-Q on the postintervention measurement. Information about completion of intervention sessions as well as frequency of teasing experiences and teasing-related distress is presented in Table 2. Descriptive information related to each of the 5 health dimensions from the EQ-5D-5L questionnaire is presented in Multimedia Appendix 1. Baseline and postintervention means for primary and secondary outcomes are presented in Table 3.

**Table 2:** Overview of the number of participants who completed each session and time spent on Young Person’s Face IT (YPF), as well as frequency of teasing experiences and teasing-related distress.

Variable			Boys		Girls		Total sample
Time spent on YPF (minutes), mean (SD)			187 (128)		232 (146)		215 (140)
**Completion of intervention sessions^a^ (boys: n=28, girls: n=43, total sample: N=71), n (%)**							
	0	18 (4)		N/A^b^		1 (1)	
	1	4 (14)		4 (9)		8 (11)	
	2	1 (4)		2 (5)		3 (4)	
	3	N/A		1 (2)		1 (1)	
	4	3 (11)		2 (5)		5 (7)	
	5	2 (7)		N/A		2 (3)	
	6	N/A		N/A		N/A	
	7	1 (4)		3 (7)		4 (6)	
	8	16 (57)		31 (72)		47 (66)	
Frequency of teasing experiences, mean (SD)			1.57 (0.52)		1.62 (0.68)		1.60 (0.62)
Teasing-related distress, mean (SD)			1.98 (1.12)		2.24 (1.29)		2.13 (1.22)

^a^Describes the number of participants who completed each session (eg, 8 participants completed only the first session, and 47 participants completed all sessions, including the booster session).

^b^N/A: not applicable.

**Table 3:** Baseline and postintervention means for primary and secondary outcomes by gender and for the total sample.

Variable	Boys, mean (SD)		Girls, mean (SD)		Total sample, mean (SD)	
	Baseline	Postintervention	Baseline	Postintervention	Baseline	Postintervention
BE-Appearance^a^	2.69 (0.78)	2.92 (0.65)	2.06 (1.01)	2.41 (0.89)	2.31 (0.97)	2.61 (0.84)
SAS-A^b^ total	35.32 (14.33)	30.11 (9.66)	48.05 (15.11)	43.58 (14.42)	43.03 (15.98)	38.27 (14.31)
FNE^c^	15.71 (7.62)	13.14 (4.95)	21.26 (7.99)	17.77 (6.38)	19.07 (8.26)	15.94 (6.25)
SAD-New^d^	12.93 (5.70)	11.39 (4.45)	18.05 (5.85)	17.07 (6.31)	16.03 (6.28)	14.83 (6.27)
SAD-General^e^	6.68 (2.82)	5.57 (1.81)	8.74 (3.81)	8.74 (3.15)	7.93 (3.58)	7.49 (3.11)
PSQ^f^ total	1.98 (0.54)	1.79 (0.38)	2.04 (0.51)	1.96 (0.47)	2.02 (0.52)	1.89 (0.44)
AFB^g^	2.02 (0.55)	1.82 (0.49)	2.02 (0.59)	2.05 (0.50)	2.02 (0.57)	1.96 (0.50)
CSB^h^	2.23 (0.64)	2.07 (0.55)	2.33 (0.61)	2.12 (0.59)	2.29 (0.62)	2.10 (0.57)
HB^i^	1.51 (0.69)	1.31 (0.41)	1.62 (0.68)	1.55 (0.56)	1.58 (0.68)	1.46 (0.52)
BILD-Q^j^	1.21 (0.30)	1.11 (0.22)	1.57 (0.54)	1.57 (0.58)	1.43 (0.49)	1.39 (0.52)
Self-rated health satisfaction measured with EQ-VAS^k^	83.75 (15.79)	88.75 (9.29)	75.47 (21.26)	74.88 (19.26)	78.73 (19.60)	80.35 (17.39)

^a^BE-Appearance: appearance esteem subscale of the Body Esteem Scale for Adolescents and Adults.

^b^SAS-A: Social Anxiety Scale for Adolescents.

^c^FNE: fear of negative evaluation (Social Anxiety Scale for Adolescents subscale).

^d^SAD-New: social avoidance and distress specific to new situations (Social Anxiety Scale for Adolescents subscale).

^e^SAD-General: social avoidance and distress in general (Social Anxiety Scale for Adolescents subscale).

^f^PSQ: Perceived Stigmatization Questionnaire.

^g^AFB: absence of friendly behavior (Perceived Stigmatization Questionnaire subscale).

^h^CSB: confused and staring behaviors (Perceived Stigmatization Questionnaire subscale).

^i^HB: hostile behavior (Perceived Stigmatization Questionnaire subscale).

^j^BILD-Q: Body Image Life Disengagement Questionnaire.

^k^EQ-VAS: EuroQol visual analog scale.

#### Bivariate Correlations

Generally, significant correlations were moderate to strong (refer to Multimedia Appendices 2 and 3). For the psychosocial variables and for boys, frequency of teasing experiences correlated strongly with teasing-related distress (*r*=0.696; *P*<.001), social anxiety and distress in general (*r*=0.54; *P*=.003), and life disengagement (*r*=0.51; *P*=.007). Depressive and anxiety symptoms correlated strongly with life disengagement (*r*=0.57; *P*=.002). For girls, frequency of teasing experiences correlated strongly with teasing-related distress (*r*=0.72; *P*<.001) but moderately with depressive and anxiety symptoms (*r*=0.35; *P*=.02), FNE (*r*=0.031; *P*=.04), and HB (*r*=0.47; *P*=.001). Depressive and anxiety symptoms correlated moderately with life disengagement (*r*=0.38; *P*=.01).

### Analyses of Predictors Related to Intervention Effects

To test our research question, backward multiple regression analyses were used to identify predictors of changes in body esteem, social anxiety, perceived stigmatization, life disengagement, and self-rated health satisfaction after adolescents’ use of YPF (refer to Multimedia Appendix 4 for details).

#### Primary Outcomes

For body esteem (BE-Appearance), age (β=.170), frequency of teasing experiences (β=.199), and time spent on YPF (β=.375) were retained in the final model, which was significant (*P*=.006), accounting for 13.9% of the variance in difference scores. For social anxiety (SAS-A), 4 different models were developed (total scale and 3 subscales). For the total scale, gender (β=–.191), frequency of teasing experiences (β=.265), depressive and anxiety symptoms (β=.230), and time spent on YPF (β=.226) were retained in the final model, which was significant (*P*=.009), accounting for 13.8% of the variance. For the subscale FNE, teasing (β=.389) and time spent on YPF (β=.261) were retained in the final model, which was significant (*P*=.001), accounting for 16.7% of the variance. For the subscale SAD-New, gender (β=–.199) and depressive and anxiety symptoms (β=.227) were retained in the final model, which was not significant (*P*=.11), accounting for 3.6% of the variance. For the subscale SAD-General, gender (β=–.270), frequency of teasing experiences (β=.252), and depressive and anxiety symptoms (β=.214) were retained in the final model, which was significant (*P*=.005), accounting for 14.3% of the variance.

#### Secondary Outcomes

For perceived stigmatization (PSQ), 4 different models were developed (total scale and 3 subscales). For the total scale (PSQ total), age (β=–.204), gender (β=–.207), frequency of teasing experiences (β=.163), and depressive and anxiety symptoms (β=.245) were retained in the final model, which was significant (*P*=.03), and accounted for 10.5% of the variance. For the subscale AFB, gender (β=–.301) and depressive and anxiety symptoms (β=.263) were retained in the final model, which was significant (*P*=.02), and accounted for 8.7% of the variance. For the subscale CSB, only age (β=–.174) was retained in the final model, which was not significant (*P*=.16), and accounted for 1.6% of the variance. For the subscale HB, age (β=–.173), gender (β=–.180), frequency of teasing experiences (β=.383), and depressive and anxiety symptoms (β=.240) were retained in the final model, which was also significant (*P*<.001), and accounted for 24.1% of the variance. For life disengagement (BILD-Q), gender (β=–.257), depressive and anxiety symptoms (β=.428), and time spent on YPF (β=.169) were retained in the final model, which was significant (*P*=.003), and accounted for 15.8% of the variance. Finally, for self-rated health satisfaction (EQ-VAS), only age (β=–.319) was retained in the final model, which was also significant (*P*=.008), and accounted for 8.8% of the variance.

## Discussion

### Principal Findings

To obtain a better understanding of which adolescents are likely to benefit from YPF, this study explored how demographic (age and gender), psychosocial (baseline frequency of teasing experiences as well as depressive and anxiety symptoms), and intervention-related (time spent on YPF) variables predicted changes in a range of outcomes. The principal findings of the study were that different combinations of demographic, psychosocial, and intervention-related variables predicted intervention effects on primary and secondary outcomes. In general, explained variance was higher in analyses that included primary outcomes than in those that included secondary outcomes. Nonetheless, it is important to note that explained variance overall was relatively low for most of our regression models (ranging from 1.6% to 24.1%), which should be taken into account when interpreting our results. The findings are discussed in more detail in the following sections.

### Comparison With Prior Work

Adolescents who reported greater baseline psychosocial distress in the form of higher frequency of teasing experiences as well as depressive and anxiety symptoms had stronger effect of YPF on primary and secondary outcomes than adolescents with lower levels of psychosocial distress. These results could indicate that adolescents who experience higher levels of psychosocial distress may benefit more from YPF than adolescents who experience relatively lower distress. Indeed, similar to our findings, studies with adolescent community samples have found that higher pretest symptoms of anxiety [48,49] and depression [48] have predicted greater reductions in posttest symptoms of anxiety after ICBT. Although speculative, it is possible that adolescents who experience higher psychosocial distress may have more potential for improvement and may be more motivated to engage with the intervention content.

Frequent experiences of different types of teasing in childhood, including teasing about appearance, have been linked to symptoms of social anxiety in adulthood [50], and adolescents who frequently experience peer victimization are also more likely to develop an anxiety disorder than nonvictimized adolescents [51]. Moreover, symptoms of depression and anxiety developed during adolescence [52] and higher psychological distress in childhood and adolescence in general [53] may persist into adulthood and increase the risk of poorer health. Hence, providing evidence-based support for adolescents with a visible difference who experience higher levels of psychosocial distress seems imperative to promote psychosocial well-being and adjustment. However, more studies are required to further investigate whether higher levels of psychosocial distress consistently predict stronger effect of YPF in adolescents with a visible difference and whether stronger intervention effects are maintained over time for this group.

This study also found that time spent on YPF predicted stronger intervention effects on primary and secondary outcomes, suggesting that adolescents who spend more time on YPF and thereby engage more with the content of the intervention achieve a higher intervention response. This aligns with the study by Williamson et al [24], which found that increased engagement with YPF (ie, number of YPF sessions completed) predicted positive changes in body esteem and social anxiety. By contrast, the number of weeks that participants spent completing YPF, irrespective of the number of sessions completed, did not predict any changes after the intervention in the study by Zelihić et al [31]. Our results are also in line with those of other studies with adolescent community samples showing that intervention engagement seems to be important for adolescents’ response to ICBT [49,54], suggesting that increased engagement allows for greater therapeutic effects. It should therefore be considered key for future studies on YPF to identify variables that may motivate adolescents to spend more time on the intervention. Some recent studies have suggested that reduced amount of text; ability to personalize the intervention and user-friendliness [55]; and involving, for example, parents or professionals [56] may improve intervention engagement and adherence. Such variables could possibly also facilitate increased engagement in the form of time spent on YPF among adolescent users of YPF.

We found that gender was significantly related to intervention effects because girls had consistently lower changes in primary and secondary outcome scores than boys. This corroborates some of the findings reported in the study by Zelihić et al [31], which included partly the same participants as in this study, where girls had higher postintervention scores on symptoms of social anxiety than boys. Combined, these results could suggest that boys benefit more from YPF than girls. Again, these results should be interpreted with caution considering that our prognostic models were limited by relatively low explained variance. Previous studies with adolescent community samples offer contrasting results on how gender relates to the effectiveness of interventions based on ICBT [48,57] because girls tend to have better effects [48]. Generally, in the context of adolescents with a visible difference, very little is still known about why and how effects from interventions such as YPF may vary by gender. Future studies should therefore further investigate whether boys truly benefit more from YPF, as well as whether other variables (eg, motivation to complete YPF and changes in the YPF content) contribute to predict girls’ intervention response.

Age did not provide a consistent picture of intervention effects. Whereas higher age predicted higher changes in body esteem, lower age predicted higher changes in perceived stigmatization and self-rated health satisfaction. Generally, studies with adolescent community samples also offer inconsistent results regarding the importance of age in predicting intervention effects of standard CBT and ICBT [58,59]. More research on the role of age as a predictor of intervention effects of YPF and other types of psychosocial support for adolescents with a visible difference is therefore needed; for instance, it could be that mental age is a better predictor than biological age.

### Clinical Implications

This study has several important implications that may guide the referral of adolescents to YPF. Our results indicate that YPF may have an increased benefit for adolescents who experience high levels of psychosocial and psychological distress because of, for example, appearance-related teasing or high levels of depressive and anxiety symptoms. However, it is important to note that YPF may not benefit all adolescents with a visible difference, and future studies should further investigate which indicators may consistently predict intervention effects. Our results also suggest that time spent on YPF matters for intervention effects, in line with previous testing of YPF [24]. Hence, adolescents referred to YPF should be encouraged to spend enough time on each session to hopefully increase therapeutic effects, as has been demonstrated in recent research [49,54]. To facilitate increased intervention engagement, adolescents using YPF could be encouraged to complete sessions that they perceive as most beneficial for their needs, instead of focusing on completing all sessions. In addition, with the adolescent’s consent or wish, parents of adolescents referred to YPF could be advised to monitor the adolescent’s progress as well as discuss key points within each session, which may strengthen parent-adolescent communication [60].

Adolescents with moderate-to-severe psychological difficulties (eg, clinical depression, eating disorders, or posttraumatic stress disorder) were excluded from this study based on the clinically informed premise that they are likely to require alternative higher-level treatment. However, only 1 participant (with concurrent diagnosis of eating disorder and clinical depression) was excluded on this basis. Given the findings from a recent study that demonstrated that YPF is safe [61], it would be interesting for future research to investigate potential intervention effects after completion of YPF for adolescents with moderate-to-severe psychological difficulties receiving psychological support elsewhere to explore whether YPF could be beneficial as an adjunct to other psychological interventions. However, the authors also underscore that YPF was designed to be a low-level intervention, targeting appearance-related and social distress in adolescents with a visible difference, and was not developed for adolescents with more severe psychological difficulties [61].

### Limitations

This study includes some limitations that need to be addressed. First, the regression models generally showed relatively low explained postintervention variance in most primary and secondary outcomes (range 1.6%-24.1%). In other words, other unknown variables, not included in our study, could contribute to explain adolescents’ overall response to YPF. As such, we encourage future studies to include additional variables (eg, incentives to complete YPF, perceptions of user-interface design, level of family support, previous history of surgery, and psychosocial support). We also need a better understanding of how adolescents’ baseline levels of body esteem, perceived stigmatization, and life disengagement prospectively predict intervention effects of YPF.

Second, there is a lack of cross-condition or condition-specific measures developed for adolescents with a wide range of visible differences that are sensitive to different stages of adolescence or aspects other than those related to body image that may influence adolescents’ adjustment [62]. However, the PSQ has been specifically developed to assess stigmatizing experiences in children, adolescents, and adults with appearance changes after burn injuries. Nonetheless, it is possible that the other measures used in this study failed to capture other central aspects that are salient to adolescents’ experiences and adjustment to having an appearance-affecting condition. We therefore encourage the development and psychometric evaluation of cross-condition measures that could be used across different studies in research with adolescents with a visible difference. Moreover, none of the included measures had undergone previous psychometric evaluations in Norwegian, a common problem in non–English-speaking countries with small populations, which also underscores the importance of interpreting our results with caution. Relatedly, although the BILD-Q underwent a translation process, back translation was not performed because of limited resources, which could have affected the quality of the translated instrument.

Third, assessing the degree of clinically important change is vital to understand how a treatment may affect a particular group of individuals [63]. However, there are no standardized procedures to assess clinically important changes, and those proposed by, for example, Jacobson and Truax [63] require available norms or aggregation of samples from different studies to establish norms to assess clinically important changes. Normative data on the measures used in this study for adolescents with a visible difference are lacking. Hence, it is not clear whether the impacts of YPF on adolescents with a visible difference represent clinically important changes.

Fourth, a larger sample size would have increased statistical power and made it possible to use more stringent statistical analyses by, for example, exploring interaction effects in our subgroups. Nonetheless, given the lack of research on web-based interventions for adolescents with a visible difference, we believe that our study provides support for the evidence base regarding the effectiveness of YPF.

Finally, we were not able to systematically control for possible negative influences of the COVID-19 pandemic on the intervention effects of YPF, mainly because participants were enrolled in the study before, during, and after COVID-19–related lockdowns and restrictions, which also differed from districts in Norway. Emerging number of studies have found that the pandemic had a significant negative impact on the mental health of many adolescents, including increased levels of social anxiety [64] and depression [65], and may have exacerbated mental health difficulties for some adolescents [66]. However, recent research also indicates that for some individuals with a visible difference, the COVID-19 pandemic may also have provided temporary relief from social pressure [67], possibly with a corresponding beneficial psychological health effect. The intervention effects of YPF on adolescents in this study could therefore have been both negatively and positively affected by the influence of the COVID-19 pandemic. Nonetheless, research on how COVID-19 may have affected the lives of adolescents with a visible difference is still scarce, and we encourage future studies to specifically control for possible influences of a factor such as the COVID-19 pandemic.

### Conclusions

This study explored predictors of the intervention effects of YPF, a web-based psychosocial intervention designed to promote adolescents’ adjustment to having a visible difference. We specifically examined how demographic, baseline psychosocial, and intervention-related variables prospectively explained postintervention improvements in body esteem, social anxiety, perceived stigmatization, life disengagement, and self-rated health satisfaction. Our results suggest that boys and adolescents with higher levels of psychosocial distress may have increased intervention effects of YPF. Our results also suggest that time spent on YPF plays a role in the intervention effects. In sum, although more studies are needed to further investigate the intervention effects of YPF and similar intervention programs, our study advances the understanding of how web-based psychosocial support may benefit adolescents who may experience challenges with adjusting to the impact of having an appearance-affecting condition.

## Appendix

Multimedia Appendix 1 Descriptive characteristics related to each of the 5 health dimensions from the EQ-5D-5L questionnaire by gender and for the total sample.

Multimedia Appendix 2 Bivariate correlations (Pearson r and 2-tailed *P* value) among all study variables for boys only.

Multimedia Appendix 3 Bivariate correlations (Pearson r and 2-tailed *P* value) among all study variables for girls only.

Multimedia Appendix 4 Selection of predictor variables using backward multiple regression.

## Abbreviations

AFB: absence of friendly behavior (Perceived Stigmatization Questionnaire subscale)
BE-Appearance: appearance esteem subscale of the Body Esteem Scale for Adolescents and Adults
BESAA: Body Esteem Scale for Adolescents and Adults
BILD-Q: Body Image Life Disengagement Questionnaire
CBT: cognitive behavioral therapy
CSB: confused and staring behaviors (Perceived Stigmatization Questionnaire subscale)
EQ-VAS: EuroQol visual analog scale
FNE: fear of negative evaluation (Social Anxiety Scale for Adolescents subscale)
HB: hostile behavior (Perceived Stigmatization Questionnaire subscale)
ICBT: internet-delivered cognitive behavioral therapy
PSQ: Perceived Stigmatization Questionnaire
RCT: randomized controlled trial
SAD-General: social anxiety and distress in general (Social Anxiety Scale for Adolescents subscale)
SAD-New: social avoidance and distress specific to new situations (Social Anxiety Scale for Adolescents subscale)
SAS-A: Social Anxiety Scale for Adolescents
SST: social skills training
YPF: Young Person’s Face IT
