# Supplementary material for: Predictors of Adolescents’ Response to a Web-Based Intervention to Improve Psychosocial Adjustment to Having an Appearance-Affecting Condition (Young Person’s Face IT): Prospective Study
Source: JMIR Form Res. 2023 Jan 18;7:e35669. doi: 10.2196/35669 (PMC9892986; doi:10.2196/35669)
Supplement: Multimedia Appendix 1 [file formative_v7i1e35669_app1.docx]

Descriptive characteristics related to each of the 5 health dimensions from the EQ-5D-5L questionnaire by gender and for the total sample.

| Variable | | | Boys | Girls | Total |
| --- | --- | --- | --- | --- | --- |
| **Self-rated health states n/N (%)** | | |  |  |  |
|  | **Mobility** | |  |  |  |
|  |  | No problems | 23/28 (82%) | 35/43 (81%) | 58/71 (82%) |
|  |  | Slight problems | 2/28 (7%) | 5/43 (12%) | 7/71 (10%) |
|  |  | Moderate problems | 1/28 (4%) | 3/43 (7%) | 4/71 (5%) |
|  |  | Severe problems | 2/28 (7%) | N/A | 2/71 (3%) |
|  |  | Extreme problems | N/A^a^ | N/A | N/A |
|  | **Self-care** | |  |  |  |
|  |  | No problems | 28/28 (100%) | 38/43 (88%) | 66/71 (93%) |
|  |  | Slight problems | N/A | 4/43 (10%) | 4/71 (6%) |
|  |  | Moderate problems | N/A | 1/43 (2%) | 1/71 (1%) |
|  |  | Severe problems | N/A | N/A | N/A |
|  |  | Extreme problems | N/A | N/A | N/A |
|  | **Usual activities** | |  |  |  |
|  |  | No problems | 21/28 (75%) | 31/43 (72%) | 52/71 (73%) |
|  |  | Slight problems | 4/28 (14%) | 10/43 (24%) | 14/71 (20%) |
|  |  | Moderate problems | 3/28 (11%) | 1/43 (2%) | 4/71 (6%) |
|  |  | Severe problems | N/A | 1/43 (2%) | 1/71 (1%) |
|  |  | Extreme problems | N/A | N/A | N/A |
|  | **Pain and discomfort** | |  |  |  |
|  |  | No problems | 18/28 (64%) | 19/43 (45%) | 37/71 (52%) |
|  |  | Slight problems | 6/28 (22%) | 15/43 (35%) | 21/71 (30%) |
|  |  | Moderate problems | 4/28 (14%) | 7/43 (16%) | 11/71 (16%) |
|  |  | Severe problems | N/A | 1/43 /2%) | 1/71 (1%) |
|  |  | Extreme problems | N/A | 1/43 (2%) | 1/71 (1%) |
|  | **Anxiety/depression** | |  |  |  |
|  |  | No problems | 18/28 (67%) | 15/43 (35%) | 33/71 (47%) |
|  |  | Slight problems | 6/28 (22%) | 15/43 (35%) | 21/71 (30%) |
|  |  | Moderate problems | 3/28 (11%) | 12/43 (28%) | 15/71 (21%) |
|  |  | Severe problems | N/A | 1/43 (2%) | 1/71 (1%) |
|  |  | Extreme problems | N/A | N/A | N/A |

^a^ Not applicable.
