# Supplementary material for: Predictors of Adolescents’ Response to a Web-Based Intervention to Improve Psychosocial Adjustment to Having an Appearance-Affecting Condition (Young Person’s Face IT): Prospective Study
Source: JMIR Form Res. 2023 Jan 18;7:e35669. doi: 10.2196/35669 (PMC9892986; doi:10.2196/35669)
Supplement: Multimedia Appendix 2 [file formative_v7i1e35669_app2.docx]

Bivariate correlations (Pearson *r* and 2-tailed *P* value) among all study variables for boys only.

| Variable^c^ | | Age | Frequency of teasing experiences | Teasing-related distress | Time | Depressive and/or anxiety symptoms | BE-Appearance | FNE | SAD-New | SAD-General | SAS-A Total | AFB | CSB | HB | PSQ Total | BILD-Q | Self-rated health state |
| --- | --- | --- | --- | --- | --- | --- | --- | --- | --- | --- | --- | --- | --- | --- | --- | --- | --- |
| **Age** | | | | | | | | | | | | | | | | | |
|  | *r* | 1 | 0.152 | –0.115 | –0.134 | 0.073 | 0.093 | 0.103 | –0.014 | –0.073 | 0.029 | –0.346 | –0.144 | –0.126 | –0.251 | 0.135 | –0.038 |
|  | *P* value | ––^d^ | .44 | .59 | .51 | .72 | .64 | .60 | .94 | .71 | .88 | .07 | .46 | .52 | .20 | .50 | .85 |
| **Frequency of teasing** | | | | | | | | | | | | | | | | | |
|  | *r* | 0.152 | 1 | 0.696^a^ | 0.046 | 0.322 | 0.295 | 0.426^b^ | –0.061 | 0.535^a^ | 0.334 | 0.172 | 0.123 | 0.355 | 0.246 | 0.507^a^ | 0.260 |
|  | *P* value | .44 | –– | P<.001 | .82 | .10 | .13 | .02 | .76 | .003 | .08 | .38 | .53 | .06 | .21 | .007 | .18 |
| **Teasing-related distress** | | | | | | | | | | | | | | | | | |
|  | *r* | –0.115 | 0.696^a^ | 1 | 0.052 | 0.362 | 0.018 | 0.426^b^ | 0.207 | 0.605^a^ | 0.443^b^ | 0.389 | 0.241 | 0.318 | 0.375 | 0.463^b^ | 0.278 |
|  | *P* value | .59 | P<.001 | –– | .81 | .08 | .93 | .03 | .32 | .001 | .03 | .05 | .25 | .12 | .07 | .02 | .18 |
|  | **Time** |  |  |  |  |  |  |  |  |  |  |  |  |  |  |  |  |
|  | *r* | –0.134 | 0.046 | 0.052 | 1 | 0.060 | 0.203 | 0.388 | 0.181 | 0.054 | 0.278 | 0.143 | 0.196 | 0.352 | 0.278 | 0.161 | 0.192 |
|  | *P* value | .51 | .82 | .81 | –– | .78 | .32 | .05 | .38 | .79 | .17 | .49 | .34 | .79 | .17 | .44 | .35 |
|  | **Depressive and/or anxiety symptoms** | | | | | | | | | | | | | | | | |
|  | *r* | 0.073 | 0.322 | 0.362 | 0.060 | 1 | 0.118 | 0.476^b^ | 0.391^b^ | 0.132 | 0.418^b^ | 0.083 | 0.210 | 0.377 | 0.265 | 0.570^a^ | –0.062 |
|  | *P* value | .72 | .10 | .08 | .78 | –– | .56 | .01 | .04 | .51 | .03 | .68 | .29 | .05 | .18 | .002 | .76 |
|  | **BE-Appearance** | | | | | | | | | | | | | | | | |
|  | *r* | 0.093 | 0.295 | 0.018 | 0.203 | 0.118 | 1 | 0.429^b^ | 0.297 | 0.321 | 0.408^b^ | 0.235 | 0.386^b^ | 0.251 | 0.396^b^ | 0.473^b^ | 0.513^a^ |
|  | *P* value | .64 | .13 | .93 | .32 | .56 | –– | .02 | .13 | .10 | .03 | .23 | .04 | .20 | .04 | .01 | .005 |
|  | **FNE** | | | | | | | | | | | | | | | | |
|  | *r* | 0.103 | 0.426^b^ | 0.426^b^ | 0.388 | 0.476^b^ | 0.429^b^ | 1 | 0.749^a^ | 0.714^a^ | 0.963^a^ | 0.453^b^ | 0.365 | 0.670^a^ | 0.588^a^ | 0.489^a^ | 0.497^a^ |
|  | *P* value | .60 | .02 | .03 | .05 | .01 | .02 | –– | P<.001 | P<.001 | P<.001 | .02 | .06 | P<.001 | .001 | .01 | .007 |
|  | **SAD-New** | | | | | | | | | | | | | | | | |
|  | *r* | –0.014 | –0.061 | 0.207 | 0.181 | 0.391^b^ | 0.297 | 0.749^a^ | 1 | 0.454^b^ | 0.857^a^ | 0.556^a^ | 0.451^b^ | 0.434^b^ | 0.602^a^ | 0.179 | 0.355 |
|  | *P* value | .94 | .76 | .32 | .38 | .04 | .13 | P<.001 | –– | .02 | P<.001 | .002 | .02 | .02 | .001 | .37 | .06 |
|  | **SAD-General** | | | | | | | | | | | | | | | | |
|  | *r* | –0.073 | 0.535^a^ | 0.605^a^ | 0.054 | 0.132 | 0.321 | 0.714^a^ | 0.454^b^ | 1 | 0.784^a^ | 0.490^a^ | 0.429^b^ | 0.516^a^ | 0.593^a^ | 0.405^b^ | 0.433^b^ |
|  | *P* value | .71 | .003 | .001 | .79 | .51 | .10 | P<.001 | .02 | –– | P<.001 | .008 | .02 | .005 | .001 | .04 | .02 |
|  | **SAS-A Total** | | | | | | | | | | | | | | | | |
|  | *r* | 0.029 | 0.334 | 0.443^b^ | 0.278 | 0.418^b^ | 0.408^b^ | 0.963^a^ | 0.857^a^ | 0.784^a^ | 1 | 0.556^b^ | 0.458^b^ | 0.631^a^ | 0.668^b^ | 0.420^b^ | 0.492^a^ |
|  | *P* value | .88 | .08 | .03 | .17 | .03 | .03 | P<.001 | P<.001 | P<.001 | –– | .002 | .01 | P<.001 | P<.001 | .03 | .008 |
|  | **AFB** | | | | | | | | | | | | | | | | |
|  | *r* | –0.346 | 0.172 | 0.389 | 0.143 | 0.083 | 0.235 | 0.453^b^ | 0.556^a^ | 0.490^a^ | 0.556^b^ | 1 | 0.292 | 0.538^a^ | 0.696^a^ | 0.199 | 0.313 |
|  | *P* value | .07 | .38 | .05 | .49 | .68 | .23 | .02 | .002 | .008 | .002 | –– | .13 | .003 | P<.001 | .032 | .10 |
|  | **CSB** | | | | | | | | | | | | | | | | |
|  | *r* | –0.144 | 0.123 | 0.241 | 0.196 | 0.210 | 0.386^b^ | 0.365 | 0.451^b^ | 0.429^b^ | 0.458^b^ | 0.292 | 1 | 0.426^b^ | 0.847^a^ | 0.487^a^ | 0.570^a^ |
|  | *P* value | .46 | .53 | .25 | .34 | .29 | .04 | .06 | .02 | .02 | .01 | .13 | –– | .02 | P<.001 | .01 | .002 |
|  | **HB** | | | | | | | | | | | | | | | | |
|  | *r* | –0.126 | 0.355 | 0.318 | 0.352 | 0.377 | 0.251 | 0.670^a^ | 0.434^b^ | 0.516^a^ | 0.631^a^ | 0.538^a^ | 0.426^b^ | 1 | 0.762^a^ | 0.524^a^ | 0.434^b^ |
|  | *P* value | .52 | .06 | .12 | .08 | .05 | .20 | P<.001 | .02 | .005 | P<.001 | .003 | .02 | –– | P<.001 | .005 | .02 |
|  | **PSQ Total** | | | | | | | | | | | | | | | | |
|  | *r* | –0.251 | 0.246 | 0.375 | 0.278 | 0.265 | 0.396^b^ | 0.588^a^ | 0.602^a^ | 0.593^a^ | 0.668^a^ | 0.696^a^ | 0.847^a^ | 0.762^a^ | 1 | 0.516^a^ | 0.594^a^ |
|  | *P* value | .20 | .21 | .07 | .17 | .18 | .04 | .001 | .001 | .001 | P<.001 | P<.001 | P<.001 | P<.001 | –– | .006 | .001 |
|  | **BILD-Q** | | | | | | | | | | | | | | | | |
|  | *r* | 0.135 | 0.507^a^ | 0.463^b^ | 0.161 | 0.570^a^ | 0.473^b^ | 0.489^a^ | 0.179 | 0.405^b^ | 0.420^b^ | 0.199 | 0.487^a^ | 0.524^a^ | 0.516^a^ | 1 | 0.453^b^ |
|  | *P* value | .50 | .007 | .02 | .44 | .002 | .01 | .01 | .37 | .04 | .03 | .32 | .01 | .005 | .006 | –– | .02 |
|  | **Self-rated health satisfaction** | | | | | | | | | | | | | | | | |
|  | *r* | –0.038 | 0.260 | 0.278 | 0.192 | –0.062 | 0.513^a^ | 0.497^a^ | 0.355 | 0.433^b^ | 0.492^a^ | 0.313 | 0.570^a^ | 0.434^b^ | 0.594^a^ | 0.453^b^ | 1 |
|  | *P* value | .85 | .18 | .18 | .35 | .76 | .005 | .007 | .06 | .02 | .008 | .10 | .002 | .02 | .001 | .02 | –– |

^a^The correlation is significant at a significance level of .01 (2-tailed).

^b^The correlation is significant at a significance level of .05 (2-tailed).

^c^Frequency of teasing=frequency of teasing about body form, body weight, and/or appearance; Teasing-related distress=Degree of upset experienced as a result of experiences of teasing about body form, body weight, and/or appearance; Time=Mean time spent on YPF sessions (1–8); BE-Appearance=BE-Appearance subscale of the Body Esteem Scale for Adolescents and Adults (BESAA); FNE=Fear of negative evaluation (SAS-A subscale); SAD-N=Social avoidance and distress specific to new situations (SAS-A subscale); SAD-G=Social avoidance and distress in general (SAS-A subscale); Total SAS-A=Total scale score of the SAS-A; AFB=Absence of friendly behavior (PSQ subscale); CSB=Confused and staring behaviors from others (PSQ subscale); HB=Hostile behavior (PSQ subscale); Total PSQ=Total scale score of the PSQ; Life disengagement=BILD-Q; Self-rated health satisfaction=EQ VAS.

^d^Not applicable.
