# Supplementary material for: Predictors of Adolescents’ Response to a Web-Based Intervention to Improve Psychosocial Adjustment to Having an Appearance-Affecting Condition (Young Person’s Face IT): Prospective Study
Source: JMIR Form Res. 2023 Jan 18;7:e35669. doi: 10.2196/35669 (PMC9892986; doi:10.2196/35669)
Supplement: Multimedia Appendix 3 [file formative_v7i1e35669_app3.docx]

Bivariate correlations (Pearson *r* and 2-tailed *P* value) among all study variables for girls only.

| Variable^c^ | | Age | Frequency of teasing experiences | Teasing-related distress | Time | Depressive and/or anxiety symptoms | BE-Appearance | FNE | SAD-New | SAD-General | SAS-A Total | AFB | CSB | HB | PSQ Total | BILD-Q | Self-rated health state |
| --- | --- | --- | --- | --- | --- | --- | --- | --- | --- | --- | --- | --- | --- | --- | --- | --- | --- |
| **Age** | | | | | | | | | | | | | | | | | |
|  | *r* | ––^d^ | 0.07 | .35^b^ | –.15 | .06 | .16 | –.18 | –.20 | –.05 | –.19 | .06 | –.20 | –.14 | –.14 | –.13 | –.46^a^ |
|  | *P* value | 1 | .68 | .03 | .34 | .69 | .32 | .26 | .20 | .77 | .23 | .69 | .20 | .36 | .37 | .41 | .002 |
| **Frequency of teasing** | | | | | | | | | | | | | | | | | |
|  | *r* | 0.07 | –– | .72^a^ | –.22 | .35^b^ | .10 | .31^b^ | .04 | .24 | .29 | .18 | –.08 | .47^a^ | .22 | .20 | –.05 |
|  | *P* value | .68 | 1 | *P*<.001 | .15 | .02 | .51 | .04 | .81 | .12 | .06 | .24 | .59 | .001 | .16 | .20 | .76 |
| **Teasing-related distress** | | | | | | | | | | | | | | | | | |
|  | *r* | 0.35^b^ | 0.72^a^ | –– | –.01 | .37^b^ | .33^b^ | .44^a^ | .07 | .35^b^ | .44^a^ | .19 | –.05 | .37^b^ | .22 | .15 | –.22 |
|  | *P* value | .03 | *P*<.001 | 1 | .95 | .02 | .04 | .006 | .68 | .03 | .006 | .25 | .75 | .02 | .19 | .38 | .18 |
| **Time** | | | | | | | | | | | | | | | | | |
|  | *r* | –0.15 | –0.22 | –.01 | –– | –.16 | .38^b^ | .14 | .04 | .06 | .19 | .05 | .12 | –.10 | .05 | .13 | –.03 |
|  | *P* value | .34 | .15 | .95 | 1 | .32 | .01 | .39 | .81 | .68 | .45 | .75 | .44 | .53 | .74 | .41 | .84 |
| **Depressive and/or anxiety symptoms** | | | | | | | | | | | | | | | | | |
|  | *r* | 0.06 | 0.35^b^ | .37^b^ | –.16 | –– | .00 | .14 | .15 | .38^b^ | .25 | .33^b^ | .01 | .34^b^ | .29 | .38^b^ | –.04 |
|  | *P* value | .69 | .02 | .02 | .32 | 1 | .99 | .38 | .34 | .01 | .11 | .03 | .93 | .03 | .06 | .01 | .80 |
| **BE-Appearance** | | | | | | | | | | | | | | | | | |
|  | *r* | 0.16 | 0.10 | .33^b^ | .38^b^ | .002 | –– | .71^a^ | .25 | .26 | .61^a^ | .16 | .39^a^ | .17 | .36^b^ | –.01 | .12 |
|  | *P* value | .32 | .51 | .04 | .01 | .99 | 1 | *P*<.001 | .11 | .10 | *P*<.001 | .32 | .01 | .27 | .02 | .93 | .45 |
| **FNE** | | | | | | | | | | | | | | | | | |
|  | *r* | –0.18 | 0.31^b^ | .44^a^ | .14 | .14 | .71^a^ | –– | .41^a^ | .39^a^ | .89^a^ | .14 | .46^a^ | .33^b^ | .44^a^ | .20 | .29 |
|  | *P* value | .26 | .04 | .006 | .39 | .38 | *P*<.001 | 1 | .006 | .01 | *P*<.001 | .36 | .002 | .03 | .003 | .20 | .06 |
| **SAD-New** | | | | | | | | | | | | | | | | | |
|  | *r* | –0.20 | 0.04 | .07 | .04 | .15 | .25 | .41^a^ | –– | .42^a^ | .71^a^ | –.01 | .23 | .21 | .19 | .09 | .24 |
|  | *P* value | .20 | .81 | .68 | .81 | .34 | .11 | .006 | 1 | .005 | *P*<.001 | .94 | .13 | .18 | .21 | .56 | .13 |
| **SAD-General** | | | | | | | | | | | | | | | | | |
|  | *r* | –0.05 | 0.24 | .35^b^ | .06 | .38^b^ | .26 | .39^a^ | .42^a^ | –– | .69^a^ | .10 | .12 | .08 | .14 | .21 | .28 |
|  | *P* value | .77 | .12 | .03 | .68 | .01 | .10 | .01 | .005 | 1 | *P*<.001 | .55 | .45 | .63 | .38 | .17 | .07 |
| **SAS-A Total** | | | | | | | | | | | | | | | | | |
|  | *r* | –0.19 | 0.29 | .44^a^ | .12 | .25 | .61^a^ | .89^a^ | .71^a^ | .69^a^ | –– | .12 | .40^a^ | .30 | .39^b^ | .22 | .34^b^ |
|  | *P* value | .23 | .06 | .006 | .45 | .11 | *P*<.001 | *P*<.001 | *P*<.001 | *P*<.001 | 1 | .46 | .008 | .05 | .01 | .15 | .03 |
| **AFB** | | | | | | | | | | | | | | | | | |
|  | *r* | 0.06 | 0.18 | .19 | .05 | .33^b^ | .16 | .14 | –.01 | .10 | .12 | –– | .09 | .58^a^ | .73^a^ | .41^a^ | –.03 |
|  | *P* value | .69 | .24 | .25 | .75 | .03 | .32 | .36 | .94 | .55 | .46 | 1 | .57 | *P*<.001 | *P*<.001 | .006 | .84 |
| **CSB** | | | | | | | | | | | | | | | | | |
|  | *r* | –0.20 | –0.08 | –.05 | .12 | .01 | .39^a^ | .46^a^ | .23 | .12 | .40^a^ | .09 | –– | .19 | .67^a^ | .18 | .19 |
|  | *P* value | .20 | .59 | .75 | .44 | .93 | .01 | .002 | .13 | .45 | .008 | .57 | 1 | .22 | *P*<.001 | .25 | .22 |
| **HB** | | | | | | | | | | | | | | | | | |
|  | *r* | –0.14 | 0.47^a^ | .37^a^ | –.10 | .34^b^ | .17 | .33^b^ | .21 | .08 | .30 | .58^a^ | .19 | –– | .76^a^ | .41^a^ | .14 |
|  | *P* value | .36 | .001 | .02 | .53 | .03 | .27 | .03 | .18 | .63 | .05 | *P*<.001 | .22 | 1 | *P*<.001 | .007 | .38 |
| **PSQ Total** | | | | | | | | | | | | | | | | | |
|  | *r* | –0.14 | 0.22 | .22 | .05 | .29 | .36^b^ | .44^a^ | .19 | .14 | .39^b^ | .73^a^ | .67^a^ | .76^a^ | –– | .44^a^ | .15 |
|  | *P* value | .37 | .16 | .19 | .74 | .06 | .02 | .003 | .21 | .38 | .01 | *P*<.001 | *P*<.001 | *P*<.001 | 1 | .004 | .35 |
| **BILD-Q** | | | | | | | | | | | | | | | | | |
|  | *r* | –0.13 | 0.20 | .15 | .13 | .38^b^ | –.01 | .20 | .09 | .21 | .22 | .41^a^ | .18 | .41^a^ | .44^a^ | –– | .18 |
|  | *P* value | .41 | .20 | .38 | .41 | .01 | .93 | .20 | .56 | .17 | .15 | .006 | .25 | .007 | 004 | 1 | .24 |
| **Self-rated health satisfaction** | | | | | | | | | | | | | | | | | |
|  | *r* | –0.46^a^ | –0.05 | –.22 | –.03 | –.04 | .12 | .29 | .24 | .28 | .34^b^ | –.03 | .19 | .14 | .15 | .18 | –– |
|  | *P* value | .002 | .76 | .18 | .84 | .80 | .45 | .06 | .13 | .07 | .03 | .84 | .22 | .38 | .35 | .24 | 1 |

^a^The correlation is significant at a significance level of .01 (2-tailed).

^b^The correlation is significant at a significance level of .05 (2-tailed).

^c^Frequency of teasing=frequency of teasing about body form, body weight, and/or appearance; Teasing-related distress=Degree of upset experienced as a result of experiences of teasing about body form, body weight, and/or appearance; Time=Mean time spent on YPF sessions (1–8); BE-Appearance=BE-Appearance subscale of the Body Esteem Scale for Adolescents and Adults (BESAA); FNE=Fear of negative evaluation (SAS-A subscale); SAD-N=Social avoidance and distress specific to new situations (SAS-A subscale); SAD-G=Social avoidance and distress in general (SAS-A subscale); Total SAS-A=Total scale score of the SAS-A; AFB=Absence of friendly behavior (PSQ subscale); CSB=Confused and staring behaviors from others (PSQ subscale); HB=Hostile behavior (PSQ subscale); Total PSQ=Total scale score of the PSQ; Life disengagement=BILD-Q; Self-rated health satisfaction=EQ VAS.

^d^Not applicable.
