# Supplementary material for: Predictors of Adolescents’ Response to a Web-Based Intervention to Improve Psychosocial Adjustment to Having an Appearance-Affecting Condition (Young Person’s Face IT): Prospective Study
Source: JMIR Form Res. 2023 Jan 18;7:e35669. doi: 10.2196/35669 (PMC9892986; doi:10.2196/35669)
Supplement: Multimedia Appendix 4 [file formative_v7i1e35669_app4.docx]

Selection of predictor variables using backward multiple regression.^a^

| Outcomes and predictors^b^ | | Full model | | | | Final model | | | |
| --- | --- | --- | --- | --- | --- | --- | --- | --- | --- |
|  | | Coefficient (B) | *P* value | 95% CI | *R*^2^_adjusted_ | Coefficient (B) | *P* value | 95% CI | *R*^2^_adjusted_ |
| **BE-Appearance** | | | | | | | | | |
|  | |  |  |  | .111 |  |  |  | .139 |
|  | Age | .064 | .147 | –0.023 to 0.152 |  | .064 | .142 | –0.022 to 0.149 |  |
|  | Gender | .001 | .995 | –0.327 to 0.329 |  |  |  |  |  |
|  | Frequency of teasing experiences | .214 | .101 | –0.043 to 0.470 |  | .206 | .087 | –0.031 to 0.443 |  |
|  | Depressive and/or anxiety symptoms | –.017 | .868 | –0.217 to .183 |  |  |  |  |  |
|  | Time spent on YPF | .002 | .002 | 0.001 to 0.003 |  |  |  |  |  |
|  |  |  |  |  |  |  |  |  |  |
| **SAS-A Total** | | | | | | | | | |
|  | |  |  |  | .134 |  |  |  | .138 |
|  | Age | –.579 | .400 | –1.945 to 0.786 |  |  |  |  |  |
|  | Gender | –3.895 | .134 | –9.016 to 1.226 |  | –4.059 | .116 | –9.153 to 1.034 |  |
|  | Frequency of teasing experiences | 4.386 | .032 | 0.382 to 8.389 |  | 4.327 | .034 | 0.337 to 8.318 |  |
|  | Depressive and/or anxiety symptoms | 2.897 | .068 | –0.225 to 6.019 |  | 2.822 | .075 | –0.288 to 5.931 |  |
|  | Time spent on YPF | .016 | .074 | –0.002 to 0.033 |  | .017 | .055 | 0.000 to 0.034 |  |
|  |  |  |  |  |  |  |  |  |  |
| **FNE** | | | | | | | | | |
|  | |  |  |  | .147 |  |  |  | .167 |
|  | Age | –.245 | .548 | –1.055 to 0.565 |  |  |  |  |  |
|  | Gender | –.635 | .677 | –3.673 to 2.402 |  |  |  |  |  |
|  | Frequency of teasing experiences | 3.415 | .006 | 1.040 to 5.789 |  | 3.803 | .001 | 1.607 to 6.000 |  |
|  | Depressive and/ or anxiety symptoms | .998 | .286 | –0.854 to 2.850 |  |  |  |  |  |
|  | Time spent on YPF | .011 | .029 | 0.001 to 0.022 |  | .011 | .024 | 0.002 to 0.021 |  |
|  |  |  |  |  |  |  |  |  |  |
| **SAD-New** | | | | | | | | | |
|  | |  |  |  | .017 |  |  |  | .036 |
|  | Age | –.216 | .375 | –0.698 to 0.267 |  |  |  |  |  |
|  | Gender | –1.481 | .107 | –3.291 to 0.328 |  | –1.402 | .116 | –3.162 to .358 |  |
|  | Frequency of teasing experiences | –.331 | .641 | –1.746 to 1.083 |  |  |  |  |  |
|  | Depressive and/or anxiety symptoms | 1.086 | .054 | –0.018 to 2.189 |  | .924 | .075 | –0.095 to 1.944 |  |
|  | Time spent on YPF | .002 | .525 | –0.004 to 0.008 |  |  |  |  |  |
|  |  |  |  |  |  |  |  |  |  |
| **SAD-General** | | | | | | | | | |
|  | |  |  |  | .134 |  |  |  | .143 |
|  | Age | –.119 | .559 | –0.522 to 0.285 |  |  |  |  |  |
|  | Gender | –1.778 | .022 | –3.291 to –0.265 |  | –1.695 | .026 | –3.177 to –0.213 |  |
|  | Frequency of teasing experiences | 1.302 | .031 | 0.119 to 2.485 |  | 1.218 | .041 | 0.052 to 2.385 |  |
|  | Depressive and/or anxiety symptoms | .813 | .083 | –0.109 to 1.736 |  | .778 | .094 | –0.137 to 1.693 |  |
|  | Time spent on YPF | .002 | .366 | –0.003 to 0.007 |  |  |  |  |  |
|  |  |  |  |  |  |  |  |  |  |
| **PSQ Total** | | | | | | | | | |
|  | |  |  |  | .112 |  |  |  | .105 |
|  | Age | –.042 | .120 | –0.095 to 0.011 |  | –.046 | .085 | –0.099 to 0.007 |  |
|  | Gender | –.188 | .062 | –0.387 to 0.010 |  | –.168 | .092 | –0.365 to 0.028 |  |
|  | Frequency of teasing experiences | .113 | .149 | –0.042 to 0.268 |  | .102 | .192 | –0.053 to 0.257 |  |
|  | Depressive and/or anxiety symptoms | .118 | .056 | –0.003 to 0.239 |  | .115 | .062 | –0.006 to 0.237 |  |
|  | Time spent on YPF | .000 | .220 | 0.000 to 0.001 |  |  |  |  |  |
|  |  |  |  |  |  |  |  |  |  |
| **AFB** | | | | | | | | | |
|  | |  |  |  | .072 |  |  |  | .087 |
|  | Age | –.021 | .533 | –0.086 to 0.045 |  |  |  |  |  |
|  | Gender | –.303 | .017 | –0.550 to –0.057 |  | –.297 | .016 | –0.537 to –0.057 |  |
|  | Frequency of teasing experiences | .091 | .349 | –0.102 to 0.284 |  |  |  |  |  |
|  | Depressive and/or anxiety symptoms | .135 | .078 | –0.016 to 0.285 |  | .150 | .035 | 0.011 to 0.289 |  |
|  | Time spent on YPF | .000 | .391 | 0.000 to 0.001 |  |  |  |  |  |
|  |  |  |  |  |  |  |  |  |  |
| **CSB** | | | | | | | | | |
|  | |  |  |  | –.024 |  |  |  | .016 |
|  | Age | –.052 | .195 | –0.132 to 0.027 |  | –.055 | .156 | –0.132 to 0.022 |  |
|  | Gender | –.051 | .736 | –0.349 to 0.248 |  |  |  |  |  |
|  | Frequency of teasing experiences | –.017 | .885 | –0.250 to 0.216 |  |  |  |  |  |
|  | Depressive and/or anxiety symptoms | .072 | .434 | –0.110 to 0.254 |  |  |  |  |  |
|  |  | .000 | .352 | –0.001 to 0.001 |  |  |  |  |  |
|  |  |  |  |  |  |  |  |  |  |
| **HB** | | | | | | | | | |
|  | |  |  |  | .237 |  |  |  | .241 |
|  | Age | –.052 | .144 | –0.122 to 0.018 |  | –.056 | .113 | –0.125 to 0.013 |  |
|  | Gender | –.227 | .088 | –0.489 to 0.035 |  | –.209 | .111 | –0.467 to 0.049 |  |
|  | Frequency of teasing experiences | .352 | .001 | 0.147 to 0.557 |  | .342 | .001 | 0.139 to 0.545 |  |
|  | Depressive and/or anxiety symptoms | .164 | .045 | 0.004 to 0.324 |  | .161 | .048 | 0.002 to 0.321 |  |
|  | Time spent on YPF | .000 | .393 | –0.001 to 0.001 |  |  |  |  |  |
|  |  |  |  |  |  |  |  |  |  |
| **Life disengagement** | |  |  |  |  |  |  |  |  |
|  | |  |  |  | .159 |  |  |  | .158 |
|  | Age | –.022 | .472 | –0.082 to 0.038 |  |  |  |  |  |
|  | Gender | –.232 | .044 | –0.457 to –0.006 |  | –.244 | .034 | –0.469 to –0.019 |  |
|  | Frequency of teasing experiences | .111 | .214 | –0.066 to 0.287 |  |  |  |  |  |
|  | Depressive and/or anxiety symptoms | .209 | .003 | 0.072 to 0.347 |  | .235 | .001 | 0.106 to 0.364 |  |
|  | Time spent on YPF | .001 | .135 | 0.000 to 0.001 |  | .001 | .142 | 0.000 to 0.001 |  |
|  |  |  |  |  |  |  |  |  |  |
| **Self-rated health satisfaction** | |  |  |  |  |  |  |  |  |
|  | |  |  |  | .059 |  |  |  | .088 |
|  | Age | –3.595 | .012 | –6.380 to –0.809 |  | –3.691 | .008 | –6.385 to –0.997 |  |
|  | Gender | –5.326 | .312 | –15.771 to 5.120 |  |  |  |  |  |
|  | Frequency of teasing experiences | 2.701 | .511 | –5.465 to 10.867 |  |  |  |  |  |
|  | Depressive and/or anxiety symptoms | –1.437 | .654 | –7.805 to 4.932 |  |  |  |  |  |
|  | Time spent on YPF | –.002 | .929 | –0.037 to 0.033 |  |  |  |  |  |

^a^Gender=Boys coded as 0 and girls as 1

^b^Frequency of teasing=frequency of teasing about body form, body weight, and/or appearance; Time spent on YPF=Mean time in minutes spent on YPF sessions (1–8); BE-Appearance subscale=BE-Appearance subscale of the Body Esteem Scale for Adolescents and Adults (BESAA); FNE=Fear of negative evaluation (SAS-A subscale); SAD-N=Social avoidance and distress specific to new situations (SAS-A subscale); SAD-G=Social avoidance and distress in general (SAS-A subscale); SAS-A Total=Total scale score of the SAS-A; AFB=Absence of friendly behavior (PSQ subscale); CSB=Confused and staring behaviors from others (PSQ subscale); HB=Hostile behavior (PSQ subscale); PSQ Total=Total scale score of the PSQ; Life disengagement=BILD-Q; Self-rated health satisfaction=EQ VAS.
